# High-density lipoprotein 3 cholesterol is a predictive factor for arterial stiffness: a community-based 4.8-year prospective study

**DOI:** 10.1186/s12944-017-0650-z

**Published:** 2018-01-05

**Authors:** Fan Wang, Xiaona Wang, Ping Ye, Ruihua Cao, Yun Zhang, Yue Qi, Dong Zhao

**Affiliations:** 1 Department of Geriatric Cardiology, Chinese PLA General Hospital, Beijing, 100853 China; 2 Department of epidemiology, An zhen Hospital Affiliated of Capital University of Medical Sciences, Beijing, 100029 China

**Keywords:** HDL3-C, arterial stiffness, prospective study

## Abstract

**Background:**

Although drug trials with niacin and cholesteryl ester transfer protein inhibitors that substantially increase high-density lipoprotein-cholesterol (HDL-C) failed to reduce the risk of coronary heart disease, HDL protection of the cardiovascular system cannot be easily denied. Hence, it may be HDL subfractions that are responsible for the long-held and consistent cardioprotective association of HDL. Arterial stiffness has been increasingly recognized as a strong predictor of subclinical vascular disease, atherosclerotic disease, and cardiovascular mortality. As the association of HDL subfractions and arterial stiffness is not well characterized, we aimed to determine the relations between these two entities in a community-based longitudinal Chinese population sample.

**Methods:**

We evaluated the associations of plasma HDL2-C and HDL3-C subfractions with arterial stiffness measured using carotid-femoral pulse wave velocity (cf-PWV) and then multivariate logistic regression in 1447 subjects (mean age 61.3 years) from a community-based population in Beijing, China.

**Results:**

After a median follow-up of 4.8 years, Pearson’s correlation analysis revealed that HDL3-C was negatively associated with follow-up cf-PWV (*r* = −0.114; *P* = 0.001), and there was no correlation between HDL2-C and follow-up cf-PWV (*r* = −0.045; *P* = 0.181). In the multivariate logistic regression analysis, each standard deviation (SD) increase in HDL3-C was associated with a 1.490-increased likelihood of the presence of follow-up cf-PWV [odds ratio (per SD increase in HDL3-C) 1.490; 95% confidence interval 1.021–1.470; *P* = 0.039), whereas there was no relation between HDL2-C and follow-up cf-PWV.

**Conclusions:**

HDL3-C subfractions were significantly and inversely associated with arterial stiffness, suggesting that HDL subfractions are likely more important than HDL-C in preventing cardiovascular disease.

## Background

The notion that high-density lipoprotein cholesterol (HDL-C) is protective against coronary heart disease (CHD) has been recognized by the Framingham study and other well-known epidemiological studies over the past five decades [1]. Drug trials with niacin and cholesteryl ester transfer protein inhibitors that substantially increased HDL-C, however, failed to reduce the risk of CHD [2], and a Mendelian randomization study did not support a causal role for HDL-C protecting CDH [3]. Recent research has found that the HDL subfractions are heterogeneous molecules with more than 70 associated proteins [4]. The function of HDL subfractions is also heterogeneous, with their different sizes, densities, charges, and chemical compositions [5]. Therefore, as HDL is a complex, molecularly heterogeneous collection of different-sized subfractions that carry out multiple functions, it seems unlikely that a single measure (i.e., the cholesterol carried in HDL) could capture its full functionality. HDL subfractions, however, might be responsible for the long-held and consistent cardioprotective associations of HDL [6].

Broadly, HDL can be distinguished into two subfractions, by density: HDL2 cholesterol (HDL2-C) and HDL3 cholesterol (HDL3-C) [7]. HDL3-C is well approximated by the sum of small and medium HDL particles (HDL-P), whereas HDL2-C correlates strongly with large HDL-P [8, 9]. There is no consensus, however, on the functions of HDL2-C and HDL3-C. Whereas some researchers have confirmed that large HDL-Ps have a protective effect on CHD, others recognized that the small, dense, protein-rich HDL-Ps display more potent atheroprotective properties than large, buoyant cholesterol-rich particles [10, 11].

Arterial stiffness has been increasingly recognized as a strong predictor of subclinical vascular disease, atherosclerotic disease, and cardiovascular mortality [12]. Arterial stiffness was assessed noninvasively via the measurement of carotid-femoral pulse wave velocity (cf-PWV), which is the “gold standard” for determining arterial stiffness, having provided the largest amount of epidemiological evidence for its predictive value for cardiovascular events [13]. Given these findings, we sought to investigate the association of HDL subfractions with arterial stiffness in a prospective follow-up analysis. Our goal in the present study was to determine whether HDL subfractions were associated with follow-up arterial stiffness, and whether the changes in HDL subfractions were associated with the changes in arterial stiffness (cf-PWVδ) in a follow-up analysis based on a longitudinal community-dwelling cohort from China.

## Methods

### Study population

The association between HDL subfractions and arterial stiffness (cf-PWV) were investigated via routine health status checkups in the population from a community in the Pingguoyuan area of Beijing, China. A total of 1680 participants were initially recruited for cross-sectional analysis between September 2007 and January 2009. These participants were followed up from February 1 to September 30, 2013 for the first phase of the study. During the follow-up period, 181 participants were lost, and 52 were excluded from the analyses because of death, resulting in a final sample size of 1447 participants (follow-up rate 89.2%). The median follow-up interval (the second phase) for the original 1447 participants was 4.8 years. During these visits, all participants were asked to complete a questionnaire survey. Demographic information, a medical history, blood pressure measurements, and anthropometric measurements were obtained. Fasting blood and urine samples were also collected.

Informed, written consent was obtained from each participant at the time of recruitment between September 2007 and January 2009. The ethics committee of the People’s Liberation Army General Hospital approved the study.

### Clinical data collection

Information on participants was collected by the questionnaire, physical examination, and laboratory measurements. A standard questionnaire that evaluated lifestyle factors, prevalent diseases, family history, and medication used at the baseline physical examination was administered using a face-to-face counselling method. The investigation was completed by physicians in the Department of Geriatric Cardiology of the People’s Liberation Army General Hospital who were trained by the research team. Height (cm) was measured using a wall-mounted measuring tape, and weight (kg) was measured using a digital scale without shoes. Systolic and diastolic blood pressures (SBP and DBP, respectively) were measured on the right arm twice in a sitting position after 5 min of rest.

### Laboratory measurements

Venous blood samples were collected by venipuncture after an overnight fast of at least 12 h. The samples were stored at −70 °C with ethylenediaminetetraacetic acid. HDL2 and HDL3 were separated by adding sodium bromide sequentially to adjust the serum samples to different densities and then ultracentrifugation performed at 453,000 *g* for 8 h at 16 °C in a Himac centrifuge with a PR80A rotor (Hitachi, Tokyo, Japan) [14]. All other lipids and the fasting glucose were measured using Roche enzymatic assays (Roche Diagnostics GmbH, Mannheim, Germany) on a Roche autoanalyser (Roche Diagnostics, Indianapolis, IN, USA). HDL-C was measured after precipitation of non-HDL cholesterol through exposure to magnesium/dextran, using the cholesterol oxidase method (Roche Diagnostics, Indianapolis, IN, USA). LDL-C was calculated using the Friedewald equation. All testing was performed in the same laboratory by well-trained personnel following the criteria of the World Health Organization Lipid Reference Laboratories.

### Assessment of arterial stiffness

Measurements of arterial stiffness were obtained in a quiet environment in the morning. All participants were asked to avoid smoking, alcohol, and caffeine for at least 12 h before the assessments of arterial properties. Arterial stiffness was assessed by automatic cf-PWV measurements using the Complior Colson device (Createch Industrie, Paris, France). PWV was measured with two strain-gauge transducers—non-invasively using a TY-306 Fukuda pressure-sensitive transducer (Fukuda Denshi Company, Tokyo, Japan)—fixed transcutaneously over the course of a pair of arteries separated by a known distance. The carotid arteries and femoral arteries (all on the right side) were used. Measurements were repeated over 10 cardiac cycles, and the mean value was used for the final analysis. PWV was calculated from the measurement of the pulse transit time and the distance travelled by the pulse between the two recording sites (measured on the surface of the body in metres) according to the following formula [15]: PWV (m/s) = distance (m)/transit time (s).

### Definition of variables

Smoking status was defined as smoking one or more cigarettes per day for at least 1 year. Body mass index (BMI) was calculated by the following equation: weight/height^2^ (kg/m^2^). The waist–hip ratio was calculated using the following equation: waist circumference (cm)/hip circumference (cm). The estimated glomerular filtration rate (eGFR) was calculated using the following Chronic Kidney Disease Epidemiology Collaboration eq. [16]:

$$ \mathrm{eGFR}=141\times \min\ {\left(\mathrm{Scr}/\kappa, 1\right)}^{\alpha}\times \max\ {\left(\mathrm{Scr}/\kappa, 1\right)}^{-1.209}\times {0.993}^{\mathrm{Age}}\times 1.018\ \left[\mathrm{if}\  \mathrm{female}\right]\times 1.159\ \left[\mathrm{if}\mathrm{black}\right] $$

where Scr is plasma creatinine (mg/dL), κ is 0.7 for women and 0.9 for men, α is −0.329 for women and −0.411 for men, min indicates the minimum of Scr/κ or 1, and max indicates the maximum of Scr/κ or 1. Hypertension was defined as a mean SBP ≥140 mmHg, mean DBP ≥90 mmHg, both, or the use of antihypertensive medication. Diabetes mellitus was defined as a fasting glucose ≥7.0 mmol/L, glucose ≥11.1 mmol/L at 2 h after an oral 75 g glucose challenge, the use of antihyperglycaemic medication, or both.

### Statistical analyses

The characteristics were expressed as the median (interquartile range) or the mean ± standard deviation (SD) for continuous variables and percentages for dichotomous variables. Follow-up cf-PWV was defined as an elevated (≥12 m/s) or normal (<12 m/s) level [17]. Differences in the baseline levels of risk factors and clinical characteristics between participants with elevated and normal cf-PWV over 4.8 years of follow-up were analysed using a t-test for continuous variables and the *χ*^2^ test for categorical variables.

Pearson’s correlation and multiple logistic regression analysis were performed to describe the correlations between the baseline HDL-C level and follow-up arterial stiffness. Forward stepwise multivariable logistic regression was performed to obtain the odds ratios (OR) and 95% confidence intervals (CI) requiring a variable with a probability value of ≤0.10 to be entered and <0.05 to remain in the model. The dependent variables were cf-PWV. Regression models were adjusted for age and sex (model 1) as well as hypertension, diabetes mellitus, current smoking, and changes in triglycerides, total cholesterol, low-density lipoprotein cholesterol (LDL-C), Fasting glucose, 2-h postload glucose (2-HpBG), systolic BP, diastolic BP, BMI, the waist–hip ratio, and/or eGFR (model 2). As necessary, HDL-C levels and other biomarkers were normalized by natural logarithm transformation.

We investigated the association of the change in HDL3-C levels with the change in arterial stiffness (cf-PWVδI vs. cf-PWVδII) using logistic regression models. The change in HDL3-C levels was expressed as HDL3-Cδ (HDL3-C_follow-up_ − HDL3-C_baseline_). The change in arterial stiffness was expressed as PWVδ (PWV_follow-up_ − PWV_baseline_). PWVδ was categorized as PWVδI (PWV_follow-up_ − PWV_baseline_ < 0) and PWVδII (PWV_follow-up_ − PWV_baseline_ ≥ 0). Forward stepwise multivariate logistic regression analysis was performed to evaluate odds ratios (OR) and 95% confidence intervals (CI). Regression models were adjusted for age and sex (model 1) as well as for hypertension, diabetes mellitus, current smoking, and changes in triglyceride, total cholesterol, LDL-C, systolic BP, diastolic BP, BMI, the waist–hip ratio, and the eGFR (model 2).

Receiver operating characteristic (ROC) curves were used to assess the ability of the baseline HDL3-C level indices to predict arterial stiffness assessed by cf-PWV.

All analyses were conducted using SPSS software for Windows, version 13.0 (SPSS, Chicago, IL, USA). We used the Bonferroni correction for multiple testing. A value of *P* < 0.05 was considered to indicate statistical significance.

## Results

### Clinical characteristics of the participants

Altogether, we included 1447 participants in the present study. The baseline characteristics of the study population according to cf-PWV groups (elevated or normal) are summarized in Table 1. The mean age (±SD) in the study was 61.30 ± 11.4 years. Older age, male sex, hypertension, diabetes mellitus, CHD, current smoking, higher SBP, higher waist, higher waist–hip ratio, higher fasting blood glucose, higher triglyceride and LDL-C levels, and lower eGFR levels were significantly associated with elevated cf-PWV.

**Table 1:** The clinical characteristics of study participants

Parameters	overall (*n* = 1447)	cf-PWV ≥12 (*n* = 571)	cf- PWV < 12 (*n* = 876)	*P* value
Age (year)	61.30 ± 11.4	65.59 ± 10.9	52.46 ± 11.5	<0.001
Male [n (%)]	601(41.53)	270(47.28)	331(37.78)	<0.001
BMI	25.41 ± 3.32	25.41 ± 3.28	25.26 ± 4.17	0.583
Systolic BP(mmHg)	128.7 ± 17.7	135.38 ± 18.37	124.37 ± 15.93	<0.001
Diastolic BP(mmHg)	77.11 ± 10.26	77.00 ± 10.92	77.18 ± 9.82	0.762
Fasting glucose (mmol/L)	5.39 ± 1.65	5.63 ± 1.87	5.21 ± 1.35	<0.001
2-HpBG(mmol/L)	6.96 ± 1.11	7.71 ± 1.10	6.47 ± 1.23	<0.001
Triglyceride(mmol/L)	1.80 ± 1.24	1.92 ± 1.27	1.74 ± 1.24	0.017
Total cholesterol (mmol/L)	5.01 ± 0.93	5.02 ± 0.97	5.01 ± 0.89	0.845
HDL2-C (mg/dL)	29.85 ± 9.13	29.69 ± 9.44	30.05 ± 9.08	0.593
HDL3-C (mg/dL)	20.98 ± 4.22	20.62 ± 4.12	21.42 ± 4.38	0.006
HDL cholesterol (mmol/L)	1.34 ± 0.42	1.30 ± 0.43	1.37 ± 0.41	0.247
LDL cholesterol (mmol/L)	2.91 ± 0.71	2.96 ± 0.73	2.87 ± 0.69	0.030
Waist-hip ratio	0.87 ± 0.05	0.88 ± 0.06	0.86 ± 0.0.07	<0.001
eGFR (ml/min)	94.2 ± 14.3	88.18 ± 14.19	98.63 ± 12.45	<0.001
Current smoking [n (%)]	380(26.26)	175(30.65)	205(23.40)	<0.001
Hypertension [n (%)]	755(52.17)	418(73.20)	337(38.47)	<0.001
Diabetes mellitus [n (%)]	302(20.87)	183(24.22)	119(18.45)	<0.001

Note: Characteristics are reported as percentages for categorical variables and means (±SD) or median (with interquartile range) for continuous variables. The study participants were divided into two groups based on the level of cf-PWV (≥12, <12). Categorical variablesare presented as counts and percentages. The values outside the parentheses are the number of subjects, and the values inside the parentheses are prevalence

BMI, body mass index; BP, blood pressure; HDL2-C, high-density lipoprotein 2 cholesterol; HDL3-C, high-density lipoprotein 3 cholesterol; LDL, low-density lipoprotein; 2-HpBG, 2-h postload glucose; eGFR, estimated glomerular filtration rate; cf-PWV, carotid-femoral pulse wave velocity

### Association of baseline HDL subfractions with follow-up arterial stiffness

**Fig. 1:**
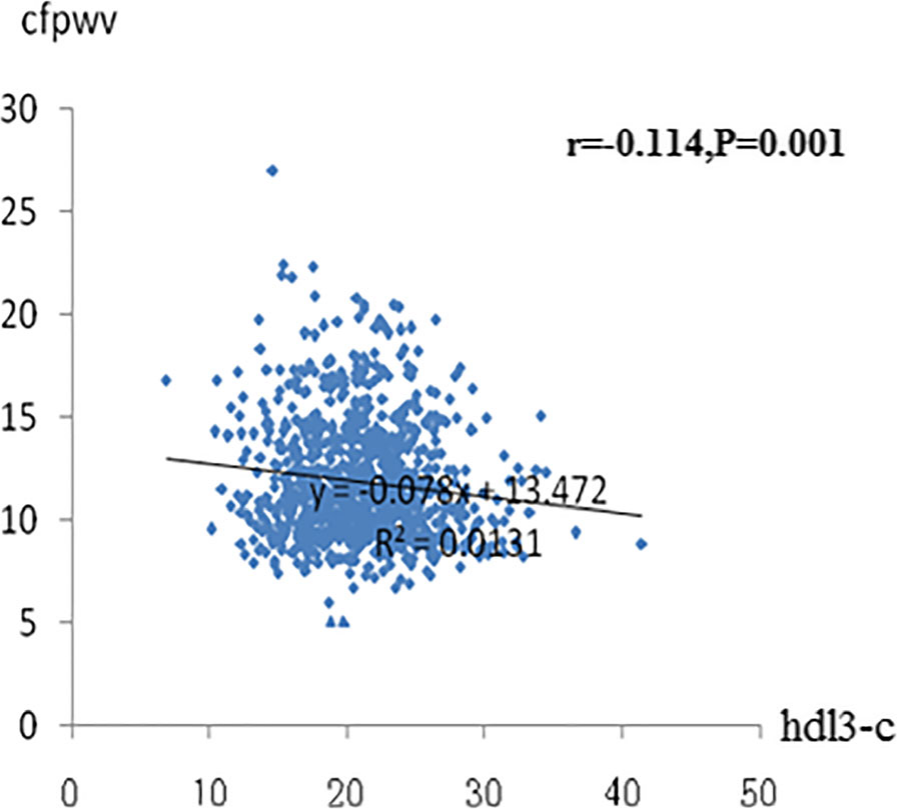
Relation between HDL3-C and cf-PWV. The Pearson’s correlation was used to describe the relationships between HDL3-C and cf-PWV. cf-PWV was negative relationship with HDL3-C in 1447 subjects. cf-PWV, carotid–femoral pulse wave velocity; HDL3-C, high-density lipoprotein 3 cholesterol; X-axis:the value of HDL3-C (mmol/L); Y-axis: the value of cf-PWV (ms^-1^); r, coefficient of Pearson’s correlation; *P* < 0.001 with statistical significance

**Fig. 2:**
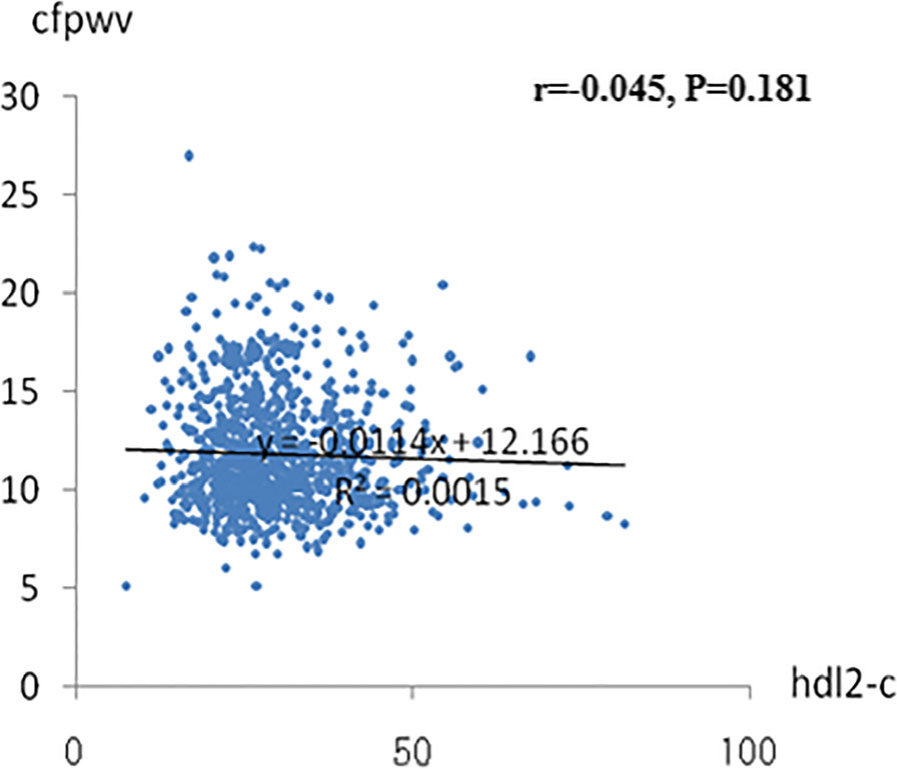
Relation between HDL2-C and cf-PWV. The Pearson’s correlation was used to describe the relationships between HDL2-C and cf-PWV. There was no relationship between cf-PWV and HDL2-C. cf-PWV, carotid–femoral pulse wave velocity; HDL2-C, high-density lipoprotein 2 cholesterol; X-axis:the value of HDL3-C(mmol/L);Y-axis: the value of cf-PWV (ms^-1^); r, coefficient of Pearson’s correlation; *P* < 0.001 with statistical significance

The association between baseline HDL subfractions as a continuous variable (natural logarithm transformed) and follow-up arterial stiffness are summarized in Fig. 1 and Fig. 2. Hypertension (*r* = 0.262; *P* < 0.001), diabetes mellitus (*r* = 0.208; *P* < 0.001), SBP (*r* = 0.259; P < 0.001), LDL-C (*r* = 0.011; *P* < 0.001), triglyceride (*r* = 0.063; P < 0.001), and 2-h postload glucose (*r* = 0.151; *P* < 00.001) were significantly and positively related to cf-PWV. HDL3-C (*r* = −0.114; *P* = 0.001) (Fig. 1) and eGFR (*r* = −0.346; P < 0.001) were inversely related to cf-PWV. Current smoking (*r* = 0.065; *P* = 0.375), BMI (*r* = −0.053; *P* = 0.181), DBP (*r* = −0.094; *P* = 0.171), total cholesterol (*r* = 0.101; *P* = 0.211), and HDL2-C (*r* = −0.045; *P* = 0.181) were not related to cf-PWV.

**Fig. 3:**
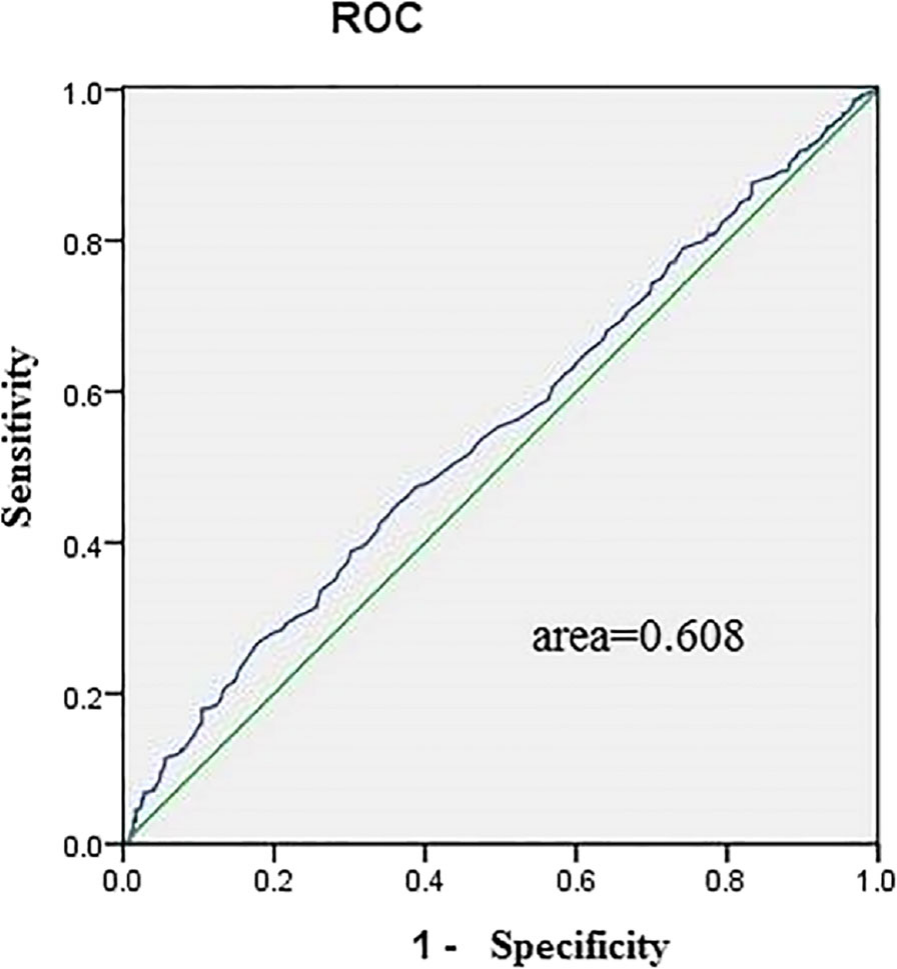
Receiver operating characteristic (ROC) curves of baseline HDL3-C indices to predict cf- PWV. ROC analysis was performed to determine the sensitivity and specificity of the value of the area under the curve (AUC)

The relation between the change in the baseline HDL3-C and the follow-up cf-PWV is presented in Table 2. The presence of follow-up cf-PWV (OR 1.045, 95% CI 1.013–1.078) (models 1 and 2) and the association of baseline HDL3-C with follow-up cf-PWV remained statistically significant. Each SD increase in HDL3-C was associated with a 1.490-increased likelihood of the presence of follow-up cf-PWV [OR (per SD increase in HDL3-C) 1.490, 95% CI 1.021–1.470; *P* = 0.039] (model 2, Table 2). In contrast, after full adjustment, the HDL2-C concentration was not significantly associated with follow-up cf-PWV (in models 1 and 2) (Table 3). The ROC curves for assessing HDL3-C indices as predictors of arterial stiffness assessed by cf-PWV are presented in Fig. 3.

**Table 2:** Logistic regression analysis for the association between baseline HDL3-C and follow-up cf-PWV

carotid-femoral PWVδ	HDL3-C		
	OR	95% CI	*P* Value
All subjects (n = 1447)			
Unadjusted	1.045	1.013~1.078	0.006
Model 1	1.437	1.050~1.966	0.023
Model 2	1.490	1.021~1.470	0.039

HDL3-C, high-density lipoprotein 3 cholesterol; PWVδ change in PWV; PWV, pulse wave velocity; OR, odds ratio; CI, confidence interval

model 1: age and gender

model 2: age, gender, hypertension, DM, current smoking, TC, TG, HDL-C, LDL-C, 2-HpBG, SBP, DBP, BMI, eGFR

**Table 3:** Logistic regression analysis for the association between baseline HDL2-C and follow-up cf-PWV

carotid-femoral PWVδ	HDL2-C		
	OR	95% CI	*P* Value
All subjects (*n* = 1447)			
Unadjusted	1.097	0.807~1.491	0.554
Model 1	1.019	0.746~1.393	0.904
Model 2	0.943	0.605~1.470	0.796

HDL2-C, high-density lipoprotein 2 cholesterol; PWVδ, change in PWV; PWV, pulse wave velocity; OR, odds ratio; CI, confidence interval

model 1: age and gender

model 2: age, gender, hypertension, DM, current smoking, TC, TG, HDL-C, LDL-C, 2-HpBG, SBP, DBP, BMI, eGFR

### Effect of change in HDL3-C on change in arterial stiffness

The relation between the change in HDL3-C concentration (HDL3-Cδ) and the change in the cf-PWV (cf-PWVδI vs. cf-PWVδII) are presented in Table 4. The presence of cf-PWVδ was not related to HDL3-Cδ in either the unadjusted or adjusted models.

**Table 4:** Logistic regression analysis for the association between change in HDL3-C and change in cf-PWV

carotid-femoral PWV δ II	HDL3-C δ		
	OR	95% CI	*P* Value
All subjects (n = 1447)			
Unadjusted	1.217	0.798~1.855	0.361
Model 1	0.959	0.579~1.540	0.862
Model 2	1.608	0.487~5.307	0.436

HDL3-C, high-density lipoprotein 3 cholesterol; HDL3-Cδ, change in HDL3-C; PWV, pulse wave velocity; PWVδII, PWV_follow-up_-PWV_baseline_ ≥ 0; OR, odds ratio; CI, confidence interval

model 1: age and gender

model 2: age, gender, hypertension, DM, current smoking, baseline carotid-femoral PWV, change in TC, change in TG, change in LDL-C, change in SBP, change in DBP, change in BMI, change in Waist–hip ratio and change in eGFR

## Discussion

This is the first study to observe the associations of HDL subfraction levels with cf-PWV in a community-based prospective sample. In this longitudinal study, we found that baseline HDL3-C levels were negatively and independently associated with follow-up cf-PWV. After full adjustment, however, HDL2-C levels were not significantly associated with follow-up cf-PWV. Our results revealed that HDL3-C may be an independent protective factor against arterial stiffness.

The cf-PWV is considered the gold standard for measuring arterial stiffness [18], which plays an important role in subclinical vascular damage and cardiovascular disease. It has an independent predictive value for all-cause mortality and cardiovascular mortality, cardiovascular disease, fatal and nonfatal coronary events, and fatal strokes. Some studies on individuals with familial hypercholesterolaemia suggest that there is a positive relation between arterial stiffness and serum lipids [19]. In the present prospective study, we analysed the correlation between the baseline HDL subfractions HDL2-C and HDL3-C and follow-up cf-PWV. After adjusting the factors, HDL3-C subfraction levels were negatively independently associated with cf-PWV, whereas this correlation was not found for HDL2-C.

HDL2-C is larger, less dense, and strongly associated with apolipoprotein A1 (apoA1), which carries most of the cholesterol reflected in HDL-C measurements. HDL3-C is well approximated by the sum of the small and medium HDL-P concentrations [20]. HDL3 carries enzymes that prevent oxidative stress and receives cholesterol from reverse cholesterol transport through the ATP-binding cassette transporter A1 mediators [21]. Earlier studies that measured the cholesterol content of HDL subclasses using density gradient ultracentrifugation suggested that the large HDL2-C subfractions are better predictors of CHD. With the availability of newer HDL measures, however, more recent studies using the same separation technique have found that smaller HDL3-C subfractions play this role [22, 23]. Chobufo Ditah et al. [24] reported that, in a cross-sectional population-based sample, small and medium-sized HDL particles are protectively associated with coronary calcification. MESA found that smaller HDL particles levels were inversely correlated with carotid intima media thickening after confounder adjustment [25]. Our findings of no statistically significant association between HDL2-C and cf-PWV suggested that HDL-2 particles afforded no protection against arterial stiffness, which is consistent with a recent report [26].

The mechanisms underlying the relation between HDL3-C subfractions and arterial stiffness are not yet fully understood. Several potential mechanisms may help explain the protection of HDL3-C subfractions regarding arterial stiffness. First, the development of atherosclerotic plaques on the arterial wall may lead to arterial stiffness. Cholesterol efflux has an inverse relation with HDL subfractions’ size and density, with the smallest HDL subfractions having the highest efficiency for cholesterol efflux from macrophage foam cells by ATP-binding cassette transporter A1 [27]. HDL3-C may be able to prevent the development of atherosclerotic plaques. Second, a previous study found that antioxidant activity and antiapoptotic actions were enriched in HDL3-C [28], and it is known that oxidative stress and apoptosis can destroy the function of arterial endothelial cells. Third, increasing numbers of studies confirmed that inflammation plays a major role in arterial stiffness. Recent work in MESA found that HDL3-C was inversely associated with non-cardiovascular inflammation-related death or hospitalization, suggesting that HDL plays an important role in the pathogenesis of numerous inflammation-related diseases [29]. In this regard, the HDL content of sphingosine-1-phosphate (S1P) and other lipids carried on HDL are important as possible molecular mediators of this anti-inflammatory effect [30]. S1P is enriched in small and dense HDL [31] and is inversely correlated with endothelial cell apoptosis [32]. That is, HDL3-C and arterial stiffness have a pathophysiological correlation.

The association between the changes in HDL3-C subfractions and those in cf-PWV was investigated in the present study. The results showed that there was no association between the changes in HDL3-C subfractions and those in cf-PWV. Therefore, we believe that the baseline HDL3-C subfractions have predictive value for arterial stiffness, but elevated HDL3-C subfractions do not alleviate it.

### Study limitations

Limitations of this study should be considered. First, these results imply that unmeasured functional aspects of HDL3-C subfractions are at least partially responsible for the HDL-C independent and inverse association of small and medium HDL-Ps and arterial stiffness. Second, the role of diet on lipids should be discussed. There were many studies reported that nutraceuticals and functional food ingredients were beneficial to vascular health and reduced the overall cardiovascular risk induced by dyslipidaemia except statins therapy [33]. Some nutraceuticals such as red wine, grape seed, and fish oil maybe have protective effect on vascular system by increasing the level of HDL-C [33]. Third, it was performed on Chinese residents from two communities in Beijing. Thus, the results may not represent Chinese individuals from other areas. Fourth, several variables with importance to cardiovascular disease in general and HDL in particular were not available and thus could not be included in analyses. Fifth, Inter and intra observer variability coefficients for PWV assessments should be considered. In order to mitigate the bias, the measure of PWV was carried out by the same physician who was fully trained by the research team and implement according to pre-approved protocol. The measurements of arterial stiffness were taken standardly and repeated over 10 cardiac cycles, and the mean value was used for the final analysis. Finally, 181 patients (10.7%) were lost to follow-up. This loss is an unavoidable limitation of epidemiological studies that may be biased toward the null hypothesis because of the loss of cases that presumably had more extreme values for the analysed variables.

### Study strengths

This study is the first analysis on the association of HDL subfractions with arterial stiffness based on a longitudinal study that used baseline and follow-up data. Thus, it differs from cross-sectional studies or cohort studies that describe only baseline HDL-C measurements.

## Conclusion

We performed analyses on participants in a prospective cohort and report that HDL3-C subfractions are significantly and inversely associated with arterial stiffness. HDL2-C subfractions are not associated with arterial stiffness, suggesting that HDL subfractions are likely more important in preventing cardiovascular and cerebrovascular disease. Future work should evaluate the specific functional aspects of HDL, including S1P activity, to identify biomarkers for clinical intervention.

## Abbreviations

2-HpBG: 2-h postload glucose
BMI: body mass index
cf-pwv: arotid–femoral pulse wave velocity
CHD: coronary heart disease
DBP: diastolic blood pressure
DM: diabetes mellitus
eGFR: estimated glomerular filtration rate
FBG: fasting blood glucose
HDL2-C: HDL2 cholesterol
HDL3-C: HDL3 cholesterol
HDL-C: high-density lipoprotein cholesterol;
HDL-P: HDL particles
LDL-C: low-density lipoprotein cholesterol
ROC: receiver operating characteristic
SBP: systolic blood
TC: total cholesterol

## References

[1] Di Angelantonio E, Sarwar N, Perry P, Kaptoge S, Ray KK, Thompson A (2009). Emerging risk factors collaboration. Major lipids, apolipoproteins, and risk of vascular disease. JAMA.

[2] Kingwell BA, Chapman MJ, Kontush A, Miller NE (2014). HDL-targeted therapies: progress, failures and future. Nat Rev Drug Discov.

[3] Voight BF, Peloso GM, Orho-Melander M, Frikke-Schmidt R, Barbalic M, Jensen MK (2012). Plasma HDL cholesterol and risk of myocardial infarction: a mendelian randomisation study. Lancet.

[4] Garcia-Rios A, Nikolic D, Perez-Martinez P, Lopez-Miranda J, Rizzo M, Hoogeveen RC (2014). LDL and HDL subfractions, dysfunctional HDL: treatment options. Curr Pharm Des.

[5] Vickers KC, Remaley AT (2014). Thematic review series: high density lipoprotein structure, function, and metabolism HDL and cholesterol: life after the divorce?. J Lipid Res.

[6] Superko HR, Pendyala L, Williams PT, Momary KM, King SB, Garrett BC (2012). High-density lipoprotein subclasses and their relationship to cardiovascular disease. J Clin Lipidol.

[7] Ito Y, Satoh N, Ishii T, Kumakura J, Hirano T. Development of a homogeneous assay for measurement of high-density lipoprotein-subclass cholesterol. Clin Chim Acta. 2014;427:86-93. 10.1016/j.cca.2013.09.009 24055774

[8] Ashmaig ME, Gupta S, McConnell JP, Warnick GR (2013). Validation of a novel homogeneous assay for of HDL3-C measurement. Clin Chim Acta.

[9] Anagnostis P, Stevenson JC, Crook D, Johnston DG, Godsland IF (2015). Effects of menopause, gender and age on lipids and high-density lipoprotein cholesterol subfractions. Maturitas.

[10] Lamarche B, Moorjani S, Cantin B, Dagenais GR, Lupien PJ, Després JP (1997). Associations of HDL2 and HDL3 subfractions with ischemic heart disease in men: prospective results from the Quebec cardiovascular study, Arterioscler. Thromb Vasc Biol.

[11] López-Olmos V, Carreón-Torres E, Luna-Luna M, Flores-Castillo C, Martínez-Ramírez M, Bautista-Pérez R, et al. Increased HDL Size and Enhanced Apo A-I Catabolic Rates Are Associated With Doxorubicin-Induced Proteinuria in New Zealand White Rabbits. Lipids. 2016;51:311-20. 10.1007/s11745-016-4120-6 26781765

[12] Ambrosino P, Lupoli R, Tortora A, Cacciapuoti M, Lupoli GA, Tarantino P (2016). Cardiovascular risk markers in patients with primary aldosteronism: a systematic review and meta-analysis of literature studies. Int J Cardiol.

[13] Vlachopoulos C, Aznaouridis K, Stefanadis C (2010). Prediction of cardiovascular events and all-cause mortality with arterial stiffness: a systematic review and meta-analysis. J Am Coll Cardiol.

[14] Hirano T, Nohtomi K, Koba S, Muroi A, Ito YA (2008). Simple and precise method for measuring HDL-cholesterol subfractions by a single precipitation followed by homogenous HDL-cholesterol assay. J Lipid Res.

[15] McEniery C, Cockcroft JR (2007). Does arterial stiffness predict atherosclerotic coronary events?. Adv Cardiol.

[16] Zhao F, Zhang L, Lu J, Guo K, Wu M, Yu H (2015). The chronic kidney disease epidemiology collaboration equation improves the detection of hyperfiltration in Chinese diabetic patients. Int J Clin Exp Med.

[17] Bai Y, Ye P, Luo L, Xiao W, Xu R, Wu H, Bai J (2011). Arterial stiffness is associated with minimally elevated high-sensitivity cardiac, troponin T levels in a community-dwelling population. Atherosclerosis.

[18] Wang XN, Ye P, Cao RH, Yang X, Xiao WK, Zhang Y (2015). Plasma Homocysteine is a predictive factor for arterial stiffness: a community-based 4.8-year prospective study. J Clin Hypertens (Greenwich).

[19] Ershova AI, Meshkov AN, Rozhkova TA, Kalinina MV, Deev AD, Rogoza AN (2016). Carotid and aortic stiffness in patients with heterozygous familial hypercholesterolemia. PLoS One.

[20] Hirano T (2014). Abnormal lipoprotein metabolism in diabetic nephropathy. Clin Exp Nephrol.

[21] de la Llera-Moya de M, Drazul-Schrader D, Asztalos BF, Cuchel M, Rader DJ (2010). The ability to promote efflux via ABCA1 determines the capacity of serum specimens with similar high-density lipoprotein cholesterol to remove cholesterol from macrophages. Arterioscler Thromb Vasc Biol.

[22] Martin SS, Khokhar AA, May HT, Kulkarni KR, Blaha MJ, Joshi PH (2015). HDL cholesterol subclasses, myocardial infarction, and mortality in secondary prevention: the lipoprotein investigators collaborative. Eur Heart J.

[23] Joshi PH, Toth PP, Lirette ST, Griswold ME, Massaro JM, Martin SS (2016). Lipoprotein investigators collaborative (LIC) study group. Association of high-density lipoprotein subclasses and incident coronary heart disease: the Jackson heart and Framingham offspring cohort studies. Eur J Prev Cardiol.

[24] Ditah C, Otvos J, Nassar H, Shaham D, Sinnreich R, Kark JD (2016). Small and medium sized HDL particles are protectively associated with coronary calcification in a cross-sectional population-based sample. Atherosclerosis.

[25] Kim DS, Li YK, Bell GA, Burt AA, Vaisar T, Hutchins PM (2016). Concentration of smaller high-density lipoprotein particle (HDL-P) is inversely correlated with carotid intima media thickening after confounder adjustment: the multi ethnic study of atherosclerosis (MESA). J Am Heart Assoc.

[26] Kontush A1, Chapman MJ (2006). Antiatherogenic small, dense HDL--guardian angel of the arterial wall?. Nat Clin Pract Cardiovasc Med.

[27] Yassine HN, Belopolskaya A, Schall C, Stump CS, Lau SS, Reaven PD (2014). Enhanced cholesterol efflux to HDL through the ABCA1 transporter in hypertriglyceridemia of type 2 diabetes. Metabolism.

[28] Kontush A, Chapman MJ (2010). Antiatherogenic function of HDL particle subpopulations: focus on antioxidative activities. Curr Opin Lipidol.

[29] Duprez DA, Otvos J, Tracy RP, Feingold KR, Greenland P, Gross MD (2015). High density lipoprotein subclasses and noncardiovascular, noncancer chronic inflammatory related events versus cardiovascular events: the multi ethnic study of atherosclerosis. J Am Heart Assoc.

[30] Tsai HC, Han MH (2016). Sphingosine-1-phosphate (S1P) and S1P signaling pathway: therapeutic targets in autoimmunity and inflammation. Drugs.

[31] Kontush A, Therond P, Zerrad A, Couturier M, Negre-Salvayre A, de Souza JA (2007). Preferential sphingosine-1-phosphate enrichment and sphingomyelin depletion are key features of small dense HDL3 particles: relevance to antiapoptotic and antioxidative activities. Arterioscler Thromb Vasc Biol.

[32] Kimura T, Sato K, Malchinkhuu E, Tomura H, Tamama K, Kuwabara A (2003). High-density lipoprotein stimulates endothelial cell migration and survival through sphingosine 1-phosphate and its receptors. Arterioscler Thromb Vasc Biol.

[33] Scicchitano P, Cameli M, Maiello M, Modesti PA, Muiesan ML, Novo S (2014). Nutraceuticals and dyslipidaemia: beyond the common therapeutics. J functional. foods.

